# Vitamin D status in children and adolescents with type 1 diabetes in a specialized diabetes care centre in Bangladesh

**DOI:** 10.1002/edm2.312

**Published:** 2021-11-12

**Authors:** Bedowra Zabeen, Jebun Nahar, Bulbul Ahmed, Samin Tayyeb, Nasreen Islam, Kishwar Azad

**Affiliations:** ^1^ Department of Paediatrics Life for a child (LFAC) & Changing Diabetes in Children (CDiC) Programme Bangladesh Institute of Research & Rehabilitation in Diabetes Endocrine & Metabolic Disorders (BIRDEM) Diabetic Association of Bangladesh Dhaka Bangladesh; ^2^ Department of Paediatrics Bangladesh Institute of Research & Rehabilitation in Diabetes Endocrine & Metabolic Disorders (BIRDEM) Dhaka Bangladesh

**Keywords:** Bangladesh, Children and adolescents, type 1 diabetes, vitamin D

## Abstract

**Objectives:**

High prevalence of vitamin D deficiency (VDD) in children and adolescents with type 1 diabetes (T1D) was found in several epidemiological studies. The current study aimed to assess the Vitamin D status in children and adolescents with T1D and to examine the influence of the disease characteristics on vitamin D status in a specialized care centre in a tertiary hospital in Bangladesh.

**Methods:**

Participants were enrolled in the cross‐sectional study at the time of a regularly scheduled visit to the CDiC Paediatric Diabetes Center in BIRDEM 2(Bangladesh Institute of Research and Rehabilitation of Diabetes Endocrine and Metabolic Disorders), a tertiary care hospital in Bangladesh. The demographic and clinical data were collected through medical records with a structured questionnaire.

**Results:**

Among sixty study participants, most had inadequate levels of vitamin D: deficiency 31 (51.7%), insufficiency 14 (23.3%) and sufficiency 15 (25.0%). Participants with vitamin D deficiency (VDD) were significantly older compared to the sufficient and insufficient group (*p* = .029), and were residing in urban areas (*p* = .036) and from higher socioeconomic status (*p* = .014). BMI was significantly higher in VDD compared to the sufficient group (*p* = .040). Although we observed higher median values of daily insulin requirements and HbA1c values in patients with VDD compared to patients with vitamin D insufficiency or vitamin D sufficiency, these differences did not reach statistical significance.

**Conclusions:**

The present study revealed that the prevalence of vitamin D deficiency and insufficiency among T1 diabetes children was very high. Future studies in large sample are required to assess hypovitaminosis D in youth with T1D and also the possible relating factors of vitamin D deficiency.

## Graphical abstract

Participants with Vitamin D deficiency (VDD) were significantly older (*p* = .029), were residing in urban areas (*p* = .036) and from higher socioeconomic status (*p* = .014) compared to the sufficient and insufficient group. The present study revealed that the prevalence of vitamin D deficiency and insufficiency among T1 diabetes children was very high in Bangladesh.

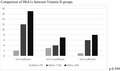

## 1 INTRODUCTION

Vitamin D deficiency (VDD) is found to be highly prevalent worldwide.^1^ In Bangladesh, the prevalence of Vitamin D deficiency is also significantly high among children.^2^, ^3^ Vitamin D deficiency represents a major health problem since it has been related to cardiovascular, inflammatory, autoimmune diseases and cancer.^4^, ^5^, ^6^, ^7^, ^8^, ^9^, ^10^, ^11^ In recent years, the extra‐skeletal effects of vitamin D have raised considerable interest since vitamin D receptor (VDR) has been found in many tissues and systems, including pancreatic β cells and immune cells.^12^, ^13^ The mechanism of transcription is the same as the skeletal actions: In the cell nucleus, VDR‐RXR heterodimer regulates the transcription of approximately 3% of the human genome.^14^, ^15^, ^16^ Several recent studies have linked vitamin D to the pathogenesis of diabetes and there is growing evidence that vitamin D can interfere with the mechanisms involved in diabetes and its complications.^1^ Vitamin D acts as a real steroid hormone and its level is influenced by estrogen status^17^ with protective action in DKD.^18^ Vitamin D supplementation has also been demonstrated to significantly reduce proteinuria and slow kidney disease progression.^19^

High prevalence of vitamin D deficiency (VDD) in children and adolescents with type 1 diabetes (T1D) was found in several epidemiological studies.^20^, ^21^ Evidence emerging that vitamin D deficiency or hypovitaminosis D may be a risk factor for T 1 D and type 2 diabetes (T2D). Some studies showed that T1D patients had lower levels of vitamin D than controls and that exposure to sunlight early in life as a source of vitamin D (VD) can prevent the development of T1D.^22^, ^23^ VD has anti‐inflammatory and immune‐modulatory effects that could influence the autoimmune pathology of T1D and may have a role in the Th1‐mediated autoimmunity against pancreatic β‐cells causing their destruction.^24^, ^25^ Vitamin D hormone has widespread effects in the immune system and the gene *CYP27B1*, which encodes the enzyme CYP1α that converts precursor 25(OH)D to 1,25(OH)D, shows association with type 1 diabetes risk.^26^, ^27^

There are significantly higher insulin requirements in T1D patients with VDD together with low insulin sensitivity, higher fasting glucose, and higher levels of glycated haemoglobin.^23^, ^24^ Although VDD is highly prevalent in children and adolescents with T1D, it is underestimated; thus, vitamin D deficiency screening and vitamin D supplementation should always be considered.^12^, ^25^ To date, there is no study on evaluating the prevalence of hypovitaminosis D in patients with type 1 diabetes in Bangladesh. The current study aimed to assess the vitamin D status in youth with T1D in a specialized care centre in a tertiary hospital in Bangladesh and to examine the influence of the disease characteristics on vitamin D status.

## 2 MATERIALS AND METHODS

Participants were enrolled in the cross‐sectional study at the time of a regularly scheduled visit to the CDiC Paediatric Diabetes Center in BIRDEM 2 hospital from January 2019 to December 2019. A structured questionnaire was used to collect data, and written informed consent was obtained from the parent and assent was obtained from the child. The diagnosis of T1DM was performed according to the ISPAD criteria and local criteria.^28^, ^29^ Patients with acute illnesses, under medication interfering with vitamin D metabolism, micro‐ or macrovascular complications were excluded during enrollment. All patients were having multiple daily dose insulin injection therapy. The demographic and clinical data including insulin requirement (IU/Kg/day) were collected through medical records.

Serum 25‐hydroxyvitamin D (25OHD) is the standard indicator of vitamin D status.^30^ Levels of 25OHD were measured with the radioimmunoassay method. The criteria used to define vitamin D deficiency (VDD) was considered with vitamin D < 11.7 ng/ml in males and <14.3 ng/ml in females, and VD insufficiency was considered with vitamin D <20 ng/ml and VD sufficiency with vitamin D ≥20 ng/ml, respectively.^31^

HbA1c was measured with high‐performance liquid chromatography standardized to the DCCT assay. HbA1c levels were categorized as <7.0% (optimal), 7.0%–9% (sub‐optimal) and >9% (poor glucose control).^32^ Height was measured by the Harpenden stadiometer and weight by using an electronic scale. Body mass index (BMI) was calculated as body weight divided by squared height (kg/m^2^) according to the growth chart.^33^

### 2.1 Statistical analyses

Data were expressed as mean ±standard deviation (SD), or percentages, or median with percentile as appropriate. For continuous variables those are not normally distributed, differences were compared using non‐parametric Mann–Whitney U test and chi‐square χ 2 test for categorical variables. Statistical significance was defined by *p* values <.05.

## 3 RESULTS

**FIGURE 1:**
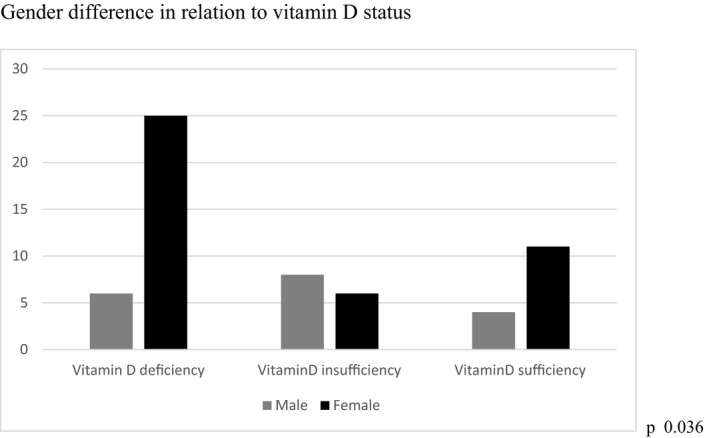
Gender difference in relation to vitamin D status

This study included 60 participants (42 females and 18 males) with T1D with a median age of 13.0[11.0–15.0] years. Most of our patients lived in urban areas (70%) in Dhaka or its vicinity. The median duration of diabetes was 2.0[1.0– 3.7] years. Median vitamin D level was 12.97[9.3–18.0] ng/ml. Among the study participants, most had inadequate levels of vitamin D deficiency 31 (51.7%), insufficiency 14 (23.3%) and sufficiency 15 (25.0%). Among the study population, male had significantly vitamin D higher median values of 14.4 [11.0–19.8ng/ml] compared to females 11.4[8.6–18.6ng/ml]. Although VDD was compared with gender, VDD was more prevalent in females (59.5%) compared to males (33.3%) [Figure 1].

**FIGURE 2:**
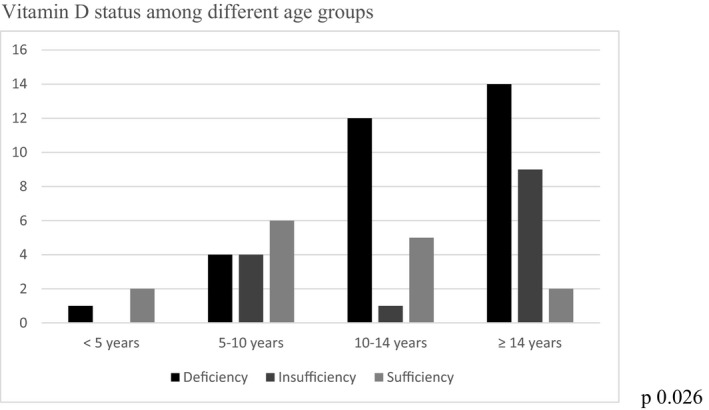
Vitamin D status among different age groups

Participants with vitamin D deficiency were significantly older compared to the sufficient and insufficient group (*p* = .029) [Table 1]. To further explore the relationship between age and vitamin D, participants were stratified into age groups and compared with vitamin D sufficiency, insufficiency or deficiency, as shown in Figure 2. VDD was significantly higher in the patients who were residing in urban areas (*p* = .036) and from higher socioeconomic status (*p* = .014) [Table 1]. There was no significant difference in the duration of diabetes between the three groups.

**TABLE 1:** Demographic and clinical characteristics between the three groups of vitamin D

Parameter	Vitamin D deficiency	Vitamin D insufficiency	Vitamin sufficiency	*p* value
Current age	14.0 [12.0–16.0]	15.5 [8.7–17.2]	10.0 [7.0–13.5]	.029
Age at onset	11.0 [9.0–13.3]	13.0 [7.0–15.0]	10.0 [4.0–12.4]	.076
Sex				
Male	6 (19.4%)	8 (57.1%)	4 (26.7%)	.036
Female	25 (80.6%)	6 (42.9%)	11 (73.6%)	
Area				
Urban	25 (59.5%)	6 (14.3%)	11 (26.2%)	.036
Rural	6 (33.3%)	8 (44.4%)	4 (22.2%)	
Socioeconomic status				
Lower	2 (6.5%)	7 (50%)	4 (26.7%)	.014
Middle	11 (35.5%)	1 (7.1%)	5 (33.3%)	
Upper	18 (58.0%)	6 (42.9%)	6 (40%)	
Diabetes duration	2.0 [1.0–4.0]	2.0 [1.0–3.0]	1.0 [1.0–3.0]	.635
BMI	22.3 [17.7–29.9]	17.1 [16.2–21.3]	16.4 [13.8–20.7]	.040
Insulin dose	29.0 [20.0–40.5]	38.0 [22.7–47.5]	24.0 [16.2–35.0]	.115
Fasting blood glucose	14.9 [9.3–18.0]	13.1 [7.9–16.1]	15.2 [11.3–23.1]	.393
HbA1c	9.1 [8.1–11.2]	9.5 [7.1–13.4]	9.8 [8.3–12.8]	.739

**FIGURE 3:**
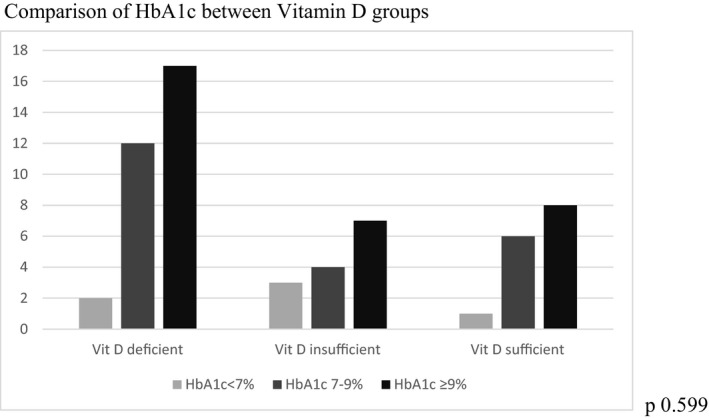
Comparison of HbA1c between Vitamin D groups

BMI was significantly higher in VDD compared to the sufficient group (*p* = .040) [Table 1]. Although there were higher median values of daily insulin requirements in VDD and VD insufficient patients compared to sufficient groups, it did not reach statistical significance (*p* = .115) [Table 1]. Median HbA1c values were high and similar in three groups (*p* = .739) [Table 1]. There was no significant difference found with the vitamin D levels. When the patients were divided into three groups according to their HbA1c% status, as being optimal (<7%), sub‐optimal glucose control (7%–9%) and poor glucose control (> 9%), there was no significant difference found with the vitamin D levels (*p* = .599) [Figure 3].

## 4 DISCUSSION

We evaluated the prevalence of vitamin D deficiency in Bangladeshi children and adolescents with T1D and found that 51.7% had VDD, 23.3% had insufficiency and 25% were VD sufficient. The high prevalence of VDD in patients with T1D was previously reported in different studies.^20^, ^21^, ^34^ Vitamin D deficiency is widespread in the South Asian population and the high prevalence of vitamin D deficiency can be explained by dark pigmentation of the skin because UV light cannot reach the appropriate layer of the skin and decreases skin synthesis of vitamin D. The low level of vitamin D in young population has been attributed mainly due to social customs, particularly the avoidance of sunlight and the breast feeding without any vitamin D supplement. However, genetic cause of VD receptor gene polymorphism may be an additional factor which was found in a study done in Egyptians patients with T1 D.^35^

In the current study, females were having more deficiency compared to males which were found in previous studies.^2^, ^36^, ^37^ This could be attributed to the fact that South Asian females are used to cover their head and body due to cultural and religious reasons; also females may have higher vitamin D requirements for bone growth during their faster pubertal growth spurt.^38^ In our study, 59.5% patients from the urban area were vitamin D deficient. Air pollution and limited outdoor activity may be responsible for this finding in the urban population.^39^

Vitamin D deficiency was more frequent in older subjects, whereas younger patients exhibited a higher prevalence of vitamin D sufficiency, a finding that was consistent with other studies.^17^, ^36^, ^37^ The present study showed no associations between vitamin D deficiency and the duration of diabetes which was observed in different studies.^21^, ^40^ On the contrary, other studies reported that VDD patients had significantly longer diabetes duration than non‐deficient patients.^20^, ^41^ The impact of BMI on the relationship between 25OHD concentration and insulin sensitivity has been proved by several studies that showed a strong correlation between overweight and hypovitaminosis D.^42^, ^43^ In our study population, we also found that BMI was significantly higher in the vitamin D deficient group. Vitamin D deficiency is especially prevalent in dark skinned children and adults living in northern latitudes, and obese children and adults.^44^

Insulin dose requirement was higher in VDD and vitamin D insufficient groups compared to sufficient group though it did not reach statistical significance. Higher insulin requirements in vitamin D deficiency and insufficient groups were found in different studies.^31^, ^40^, ^44^ The lower insulin requirements in the sufficient group may be explained by the fact that vitamin D sufficient subjects may have increased insulin sensitivity compared to VDD patients.^43^ Higher HbA1c values were found in all three groups which may implicate that VD alone may have an insignificant role in improving glycemic control in patients with T1D.^42^ Although significant glycaemic improvement after vitamin D supplementation in T1D patients with VDD was observed in some studies, there was also no significant difference observed in different studies. To date, few studies analysed the role of vitamin D treatment on glycemic control in T1DM and their results are still conflicting.^31^, ^42^, ^46^, ^47^, ^48^ Ordooei et al. and Savastio S et al. found that vitamin D administration leads to a decrease of fasting blood sugar and HbA1c levels in children and adolescents with T1 D,^45^, ^49^ whereas other studies showed no significant reduction of HbA1c after 3 months of vitamin D supplementation in children and adolescents with T1DM.^31^, ^50^

We have some limitations in our present study. This current study was limited by the fact that it was a retrospective data collection with a relatively small number of participants. It was also limited to observed associations. In addition, possible factors influencing vitamin D status (e.g. intake of supplements, lifestyle variables and dietary habits) were not investigated.

## 5 CONCLUSIONS

The present study revealed that the prevalence of vitamin D deficiency and insufficiency among T1 D children was very high. There was female preponderance and a large number of patients were from urban areas. Moreover, VDD was significantly more common in older children and participants who had high BMI, but there was no association with HbA1c. Future studies in large sample are required to assess hypovitaminosis D in youth with T1D and also the possible relating factors of vitamin D deficiency in this population.

## CONFLICTS OF INTEREST

The authors declare that they have no potential conflicts of interest relevant to this article.

## AUTHOR CONTRIBUTIONS

Authors BZ, JN and KA conceptualized and designed the study. BZ prepared the first draft of the manuscript. All authors have contributed to manuscript revisions and read the manuscript. BZ, JN and KA approved the final manuscript.

## Data Availability

n/a.
